# Transmission and Infectious SARS-CoV-2 Shedding Kinetics in Vaccinated and Unvaccinated Individuals

**DOI:** 10.1001/jamanetworkopen.2022.13606

**Published:** 2022-05-24

**Authors:** Jiwon Jung, Ji Yeun Kim, Heedo Park, Sunghee Park, Joon Seo Lim, So Yun Lim, Seongman Bae, Young-Ju Lim, Eun Ok Kim, Jineui Kim, Man-Seong Park, Sung-Han Kim

**Affiliations:** 1 Department of Infectious Diseases, Asan Medical Center, University of Ulsan College of Medicine, Seoul, South Korea; 2 Office for Infection Control, Asan Medical Center, Seoul, South Korea; 3 Department of Microbiology, Institute for Viral Diseases, Biosafety Center, College of Medicine, Korea University, Seoul, South Korea; 4 Clinical Research Center, Asan Institute for Life Sciences, Asan Medical Center, Seoul, South Korea

## Abstract

**Importance:**

Data are limited on whether patients with breakthrough COVID-19 infection have the potential to significantly contribute to the spread of SARS-CoV-2.

**Objective:**

To compare the secondary attack rate and infectious viral shedding kinetics of SARS-CoV-2 between fully vaccinated individuals (breakthrough infection group) and partially or unvaccinated individuals (nonbreakthrough infection group).

**Design, Setting, and Participants:**

This cohort study assessed secondary transmission by analyzing the epidemiologic data of health care workers, inpatients, and caregivers diagnosed with COVID-19 during hospitalization or residence in a tertiary care hospital between March 1, 2020, and November 6, 2021. To evaluate viral shedding kinetics, the genomic RNA of SARS-CoV-2 was measured using polymerase chain reaction and performed virus culture from daily saliva samples of individuals with mild COVID-19 infected with the Delta variant who were isolated in a community facility in Seoul, South Korea, between July 20 and August 20, 2021.

**Exposures:**

COVID-19 vaccination.

**Main Outcomes and Measures:**

The secondary attack rate and infectious viral shedding kinetics according to COVID-19 vaccination status.

**Results:**

A total of 173 individuals (median [IQR] age, 47 [32-59] years; 100 female [58%]) with COVID-19 were included in the secondary transmission study, of whom 50 (29%) had a breakthrough infection. Secondary transmission was significantly less common in the breakthrough infection group than in the nonbreakthrough infection group (3 of 43 [7%] vs 29 of 110 [26%]; *P* = .008). In the viral shedding kinetics study, 45 patients (median age, 37 years [IQR, 25-49 years]; 14 female [31%]) infected with the Delta variant were included, of whom 6 (13%) were fully vaccinated and 39 (87%) were partially or unvaccinated. Although the initial genomic viral load was comparable between the 2 groups, viable virus in cell culture was detected for a notably longer duration in partially vaccinated (8 days after symptom onset) or unvaccinated (10 days after symptom onset) individuals compared with fully vaccinated individuals (4 days after symptom onset).

**Conclusions and Relevance:**

In this cohort study, although the initial genomic viral load was similar between vaccinated and unvaccinated individuals, fully vaccinated individuals had a shorter duration of viable viral shedding and a lower secondary attack rate than partially vaccinated or unvaccinated individuals. Data from this study provide important evidence that despite the possibility of breakthrough infections, COVID-19 vaccinations remain critically useful for controlling the spread of SARS-CoV-2.

## Key Points

**Question:**

Do individuals fully vaccinated against COVID-19 have a shorter duration of viable SARS-CoV-2 viral shedding and a lower rate of secondary transmission than in partially vaccinated or unvaccinated individuals?

**Findings:**

In this cohort study of 173 health care workers, inpatients, and guardians and 45 participants in a community facility, secondary transmission of SARS-CoV-2 was significantly less common, and viable virus was detected for a shorter duration in fully vaccinated individuals than in partially vaccinated or unvaccinated individuals.

**Meaning:**

Fully vaccinated individuals had a shorter duration of viable viral shedding and a lower rate of secondary transmission than partially vaccinated or unvaccinated individuals.

## Teaser

This cohort study compares the secondary attack rate and infectious viral shedding kinetics of SARS-CoV-2 in fully vaccinated vs partially vaccinated and unvaccinated individuals.

## Introduction

Vaccination is the key strategy for controlling the COVID-19 pandemic. However, breakthrough infections of SARS-CoV-2 in fully vaccinated persons are occurring because of the emergence of the Delta variant (B.1.617.2) and waning of vaccine-induced immunity.^1,2^ Since July 2021, the Delta variant has emerged in South Korea and quickly became the dominant strain (9.9% in June, 61.5% in July, and 94.3% in August).^3^

There is a paucity of clinical data on the transmission risk and viral load kinetics between breakthrough infection in fully vaccinated individuals and nonbreakthrough infection, defined as SARS-CoV-2 infection in nonvaccinated or partially vaccinated individuals. Infectious viral shedding kinetics and epidemiologic data about secondary transmission between vaccinated and unvaccinated individuals with SARS-CoV-2 are essential to determine whether patients with breakthrough infection have the potential to significantly contribute to the spread of SARS-CoV-2.

In this study, we used 2 cohorts to separately determine the transmissibility of COVID-19 according to vaccination status (ie, fully vaccinated vs partially or unvaccinated). Cohort 1 included health care workers (HCWs) and inpatients and/or their caregivers or guardians in the ward of a tertiary care hospital in Seoul, South Korea, who were diagnosed with COVID-19 (during hospitalization or residence in cases of inpatient and caregiver or guardian) from March 1, 2020, to November 6, 2021; in this cohort, we aimed to compare the clinical characteristics and proportion of individuals who caused secondary infection according to vaccination status. Cohort 2 included individuals with mild COVID-19 infected with the B.1.617.2 (Delta) variant who were isolated in a community facility in Seoul, South Korea, between July 20 and August 20, 2021; in this cohort, we compared the daily viral shedding kinetics in saliva samples by genomic RNA polymerase chain reaction (PCR) and virus culture according to vaccination status.

## Methods

This cohort study included a secondary transmission study (cohort 1) and a viral kinetics shedding study (cohort 2). The methods for the identification of the Delta variant, real-time genomic PCR, and cell culture are provided in eTable 1 in the Supplement. The institutional review board of Asan Medical Center evaluated and approved the medical, scientific, and ethical aspects of our study protocol concerning cohort 1. Informed consent was waived by the ethics committee of the Asan Medical Center because this is a retrospective study that collects and analyzes medical record information that has already been obtained during epidemiologic investigation, and even if consent is not obtained the rights and welfare of the research participants are not affected. The study protocols concerning cohort 2 were approved by the institutional review board of Asan Medical Center, and all participants provided written informed consent. The design and analysis of both cohort studies followed the Strengthening the Reporting of Observational Studies in Epidemiology (STROBE) reporting guideline.

### Cohort 1: Secondary Transmission According to Vaccination Status

This study was performed at Asan Medical Center, a 2700-bed tertiary care hospital in Seoul, South Korea. The infection control team in our hospital traced closed contacts of all HCWs, inpatients, and caregivers with confirmed COVID-19, and secondary transmitted cases were identified by epidemiologic investigation. Response to a nosocomial case of COVID-19 in HCWs, inpatients, or caregivers was performed as previously reported.^4^ Detailed epidemiologic investigation, hospital policy for screening for COVID-19 cases, and the vaccination program for HCWs are given in the eMethods in the Supplement.

Data on demographic characteristics, vaccination history, date of COVID-19 diagnosis, presence of symptoms of COVID-19 at diagnosis, symptom-onset date, lowest cycle threshold (Ct) value of genes from PCR at diagnosis, and secondary transmission were retrospectively reviewed using electronic medical records or epidemiologic investigation records. Clinical characteristics and the proportion of individuals associated with nosocomial secondary infections were compared according to COVID-19 vaccination history. Because the Delta variant had emerged in South Korea in July 2021, we performed a subgroup analysis for individuals who had been diagnosed since July 2021.

### Cohort 2: Viral Shedding Kinetics of the Delta Variant According to Vaccination Status

Mildly symptomatic patients with COVID-19 in South Korea were isolated at nonhospital community facilities. Patients were monitored for at least 10 days after PCR diagnosis, and patients who needed further evaluation or medical care were transferred to nearby hospitals. According to government health policies, patients whose symptoms improved or no longer worsened at 10 days after diagnosis were discharged from the facility.

Patients with mild COVID-19 who were older than 18 years were recruited between July 20 and August 20, 2021, at a designated nonhospital community facility in Seoul, South Korea. Chest radiography was performed on the day after admission to screen for abnormalities on chest imaging. Patients who agreed to daily saliva sample collection were enrolled in this study as cohort 2.

Self-collected saliva samples were obtained from the patients starting from the day of study enrollment until the day of discharge. Each day, patients collected a 2-mL volume of saliva into an airtight container provided after they agreed to participate in the study. Patients were asked to avoid food, water, and teeth brushing for at least 30 minutes before sample collection. Saliva samples were picked up within an hour by the medical staff and transported to a designated laboratory, where they were aliquoted and stored at −80 °C until use.

### Statistical Analysis

Categorical variables were analyzed using the χ^2^ test or Fisher exact test, as appropriate. Continuous variables were analyzed using a 2-tailed *t* test or Mann-Whitney *U* test, as appropriate. Linear regression analysis was used for the trend of Ct values from vaccination to diagnosis. Survival analysis was performed using the Kaplan-Meier plot and log-rank test to estimate the negative conversion rate of PCR. Nonparametric maximum likelihood estimation was used to estimate the proportion of the viral shedding in terms of viral culture, owing to interval censoring data. General linear modeling was used to adjust for the autocorrelation on the individual level when showing the trend in viral loads according to time after symptom onset. All tests of significance were 2-tailed, and *P* < .05 was considered statistically significant. Data were analyzed using SPSS Statistics for Windows, version 23.0 (IBM Corp) or R, version 4.0.4 (R Project for Statistical Computing).

## Results

### Cohort 1: Secondary Attack Rate in Fully Vaccinated and Partially or Unvaccinated Individuals

**Figure 1.**
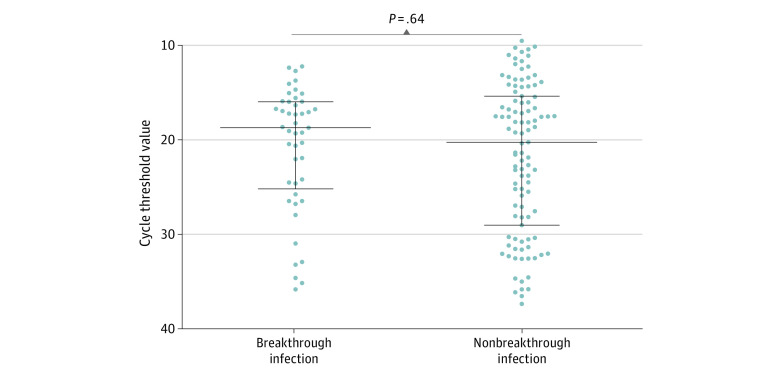
Cycle Threshold Values at Diagnosis in Cases of Breakthrough and Nonbreakthrough Infection in Cohort 1 Long horizontal lines indicate the median, and short horizontal lines indicate the IQR. No significant differences were found in the median (IQR) cycle threshold value at diagnosis in cases of breakthrough infection and nonbreakthrough infection (19 [16–24] vs 20 [15–29]; *P* = .64).

**Figure 2.**
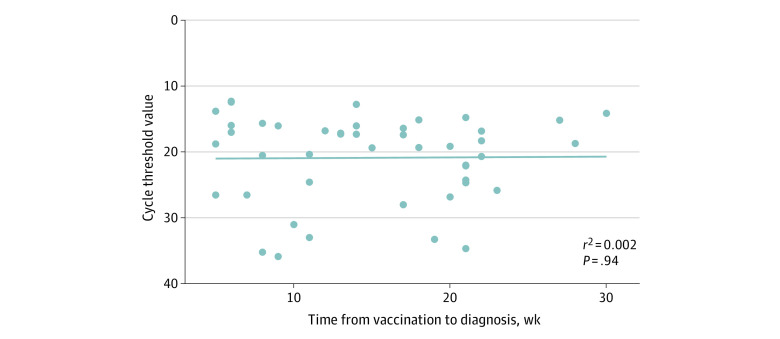
Association Between Cycle Threshold Value at Diagnosis and Weeks From Second Vaccination to Diagnosis The line represents the simple linear regression line. No significant association was found between the cycle threshold value and weeks from vaccination to diagnosis (*P* = .94 by linear regression).

A total of 173 individuals (106 HCWs, 40 inpatients, and 27 guardians or caregivers) with COVID-19 were included in cohort 1. The median age was 47 years (IQR, 32-59 years); 100 were female (58%) and 73 were male (42%). None of the individuals in this cohort had history of previous infection, and 50 (29%) had a breakthrough infection. The median time from second vaccination to diagnosis was 98 days (IQR, 57-143 days) (Table 1). Forty individuals with a breakthrough infection (80%) had received the ChAdOx-nCoV-1 vaccine. Health care workers were more common in the breakthrough infection group than in the nonbreakthrough infection group (42 [84%] vs 64 [52%]; *P* < .001). There were no significant differences in the proportion of individuals who were symptomatic at diagnosis (38 [76%] vs 82 [67%]; *P* = .23), median (IQR) time from symptom onset to diagnosis (1 [0-1] vs 1 [0-3] days; *P* = .06), and median (IQR) Ct value at diagnosis (19 [16-24] vs 20 [15-29] days; *P* = .64) between the 2 groups (Figure 1). However, among individuals who had been diagnosed without being quarantined and had the potential to transmit the virus in the hospital, the secondary nosocomial transmission rate was lower in the breakthrough infection group than in the nonbreakthrough infection group (3 of 43 [7%] vs 29 of 110 [26%], *P* = .008). There was no association between the Ct value and weeks from vaccination to diagnosis (Figure 2). When we performed the analysis with 3 groups (fully vaccinated, partially vaccinated, and nonvaccinated individuals), the results were similar to those from the analysis for breakthrough infection and nonbreakthrough infection (eTable 2 in the Supplement).

**Table 1.** Characteristics of Individuals With Breakthrough Infection and Nonbreakthrough Infection in Cohort 1 During the Entire Study Period (March 1, 2020, to November 6, 2021)

Characteristic	Breakthrough infection (n = 50)	Nonbreakthrough infection (n = 123)^a^	*P* value
Age, median (IQR), y	46 (31-59)	47 (33-59)	.80
Sex, No. (%)			
Male	23 (46)	50 (41)	.52
Female	27 (54)	73 (59)	
Participant type, No. (%)			
Health care worker	42 (84)	64 (52)	<.001
Inpatient	7 (14)	33 (27)	
Guardian or caregiver	1 (2)	26 (21)	
Time from second vaccination to diagnosis, median (IQR), d	98 (57-143)	NA	NA
Type of COVID-19 vaccine, No. (%)			
ChAdOx nCoV-19	40 (80)	NA	NA
BNT162b2	5 (10)	NA	
mRNA-1273	2 (4)	NA	
Heterologous^b^	2 (4)	NA	
Other^c^	1 (2)	NA	
Symptomatic at diagnosis, No. (%)	38 (76)	82 (67)	.23
Time from symptom onset to diagnosis, median (IQR), d	1 (0-1)	1 (0-3)	.06
Ct value at diagnosis, median (IQR)	19 (16-24)^d^	20 (15-29)^e^	.64
Nosocomial secondary transmission, No./total No. (%)^f^	3/43 (7)	29/110 (26)	.008

Abbreviations: Ct, cycle threshold; mRNA, messenger RNA; NA, not applicable.

^a^ Including 15 partially vaccinated individuals.

^b^ ChAdOx1 nCoV-19 followed by BNT162b2.

^c^ rAD26 and rAd5 vector–based vaccine.

^d^ Including 45 individuals who underwent SARS-CoV-2 testing at our hospital; Ct values were unavailable in the remaining 5 individuals.

^e^ Including 103 individuals who underwent SARS-CoV-2 testing at our hospital; Ct values were unavailable in the remaining 20 individuals.

^f^ Excluding individuals who were diagnosed during quarantine (7 in the breakthrough infection group and 13 in the nonbreakthrough infection group).

We performed a subgroup analysis in individuals who had been diagnosed since July 2021, when the Delta variant started becoming the dominant strain in South Korea (Table 2). During this period, 49 cases of breakthrough infection and 34 cases of nonbreakthrough infection occurred. Health care workers were more common in the breakthrough infection group (42 [86%] vs 18 [53%]; *P* = .003). No significant differences were found in the proportion of individuals who were symptomatic at diagnosis (38 [76%] vs 21 [62%]; *P* = .23) and median (IQR) time from symptom onset to diagnosis (1 [0-2] vs 1 [0-2] days; *P* = .21); however, the median (IQR) Ct value at diagnosis was significantly lower in the breakthrough infection group than in the nonbreakthrough infection group (19 [16-26] vs 25 [18-32]; *P* = .04). In addition, among those who were diagnosed without being quarantined, the secondary attack rate was significantly lower in the breakthrough infection group than in the nonbreakthrough infection group (3 of 43 [7%] vs 7 of 28 [25%]; *P* = .04).

**Table 2.** Characteristics of Individuals With Breakthrough Infection and Nonbreakthrough Infection in Cohort 1 After the Emergence of the Delta Variant (July 1 to November 6, 2021)

Characteristic	Breakthrough infection (n = 49)	Nonbreakthrough infection (n = 34)^a^	*P* value
Age, median (IQR), y	43 (31-59)	44 (32-56)	.65
Sex			
Male	22 (45)	10 (29)	.15
Female	27 (55)	24 (71)	
Participant type, No. (%)			
Health care worker	42 (86)	18 (53)	.003
Patient	6 (12)	10 (29)	
Guardian or caregiver	1 (2)	6 (18)	
Time from second vaccination to diagnosis, median (IQR), d	98 (57-143)	NA	NA
Type of COVID-19 vaccine, No. (%)			
ChAdOx1 nCoV-19	40 (82)	NA	NA
BNT162b2	5 (10)	NA	
mRNA-1273	2 (4)	NA	
Heterologous^b^	2 (4)	NA	
Symptomatic at diagnosis, No. (%)	37 (76)	21 (62)	.23
Time from symptom onset to diagnosis, median (IQR)	1 (0-2)	1 (0-2)	.21
Ct value at diagnosis, median (IQR)	19 (16-26)^c^	25 (18-32)^d^	.04
Nosocomial secondary transmission, No./total No. (%)^e^	3/43 (7)	7/28 (25)	.04

Abbreviations: Ct, cycle threshold; mRNA, messenger RNA; NA, not applicable.

^a^ Including 14 partially vaccinated individuals.

^b^ ChAdOx1 nCoV-19 followed by BNT162b2.

^c^ Including 44 individuals who underwent SARS-CoV-2 testing at our hospital; Ct values were unavailable in the remaining 5 individuals.

^d^ Including 28 individuals who underwent SARS-CoV-2 testing at our hospital; Ct values were unavailable in the remaining 6 individuals.

^e^ Excluding individuals who were diagnosed during quarantine (6 in the breakthrough infection group and 6 in the nonbreakthrough infection group).

We performed another subgroup analysis for secondary transmission rate stratified into HCWs and non-HCWs (eTable 3 in the Supplement). The secondary attack rate was numerically lower in the breakthrough infection group than in the nonbreakthrough infection group in both HCWs (2 of 37 [5%] vs 9 of 58 [16%]; *P* = .19) and non-HCWs (1 of 6 [17%] vs 20 of 52 [38%]; *P* = .40), albeit without statistical significance.

### Cohort 2: SARS-CoV-2 Delta Variant Shedding Kinetics Between Fully Vaccinated and Partially Vaccinated or Unvaccinated Individuals

A total of 596 patients were isolated at the facility during the study period. Among them, 538 patients were 18 years or older, and 169 adult patients initially consented to participate in the study (eFigure 1 in the Supplement). Of these patients, 156 submitted saliva samples, and the mutation kit results were inconclusive or undetected in 15 patients. Of the remaining 141 patients, 108 (77%) had been infected with the Delta variant, whereas 33 (23%) had been infected with non-Delta variants, including the Alpha variant (3 patients), untypable variants with E484K (26 patients) and L452R (1 patient), and original type (3 patients). Of the 108 patients infected with the Delta variant, 47 (44%) provided daily saliva samples and were included in the viral shedding analysis. However, viral loads were not detected in 2 patients, and they were excluded.

**Figure 3.**
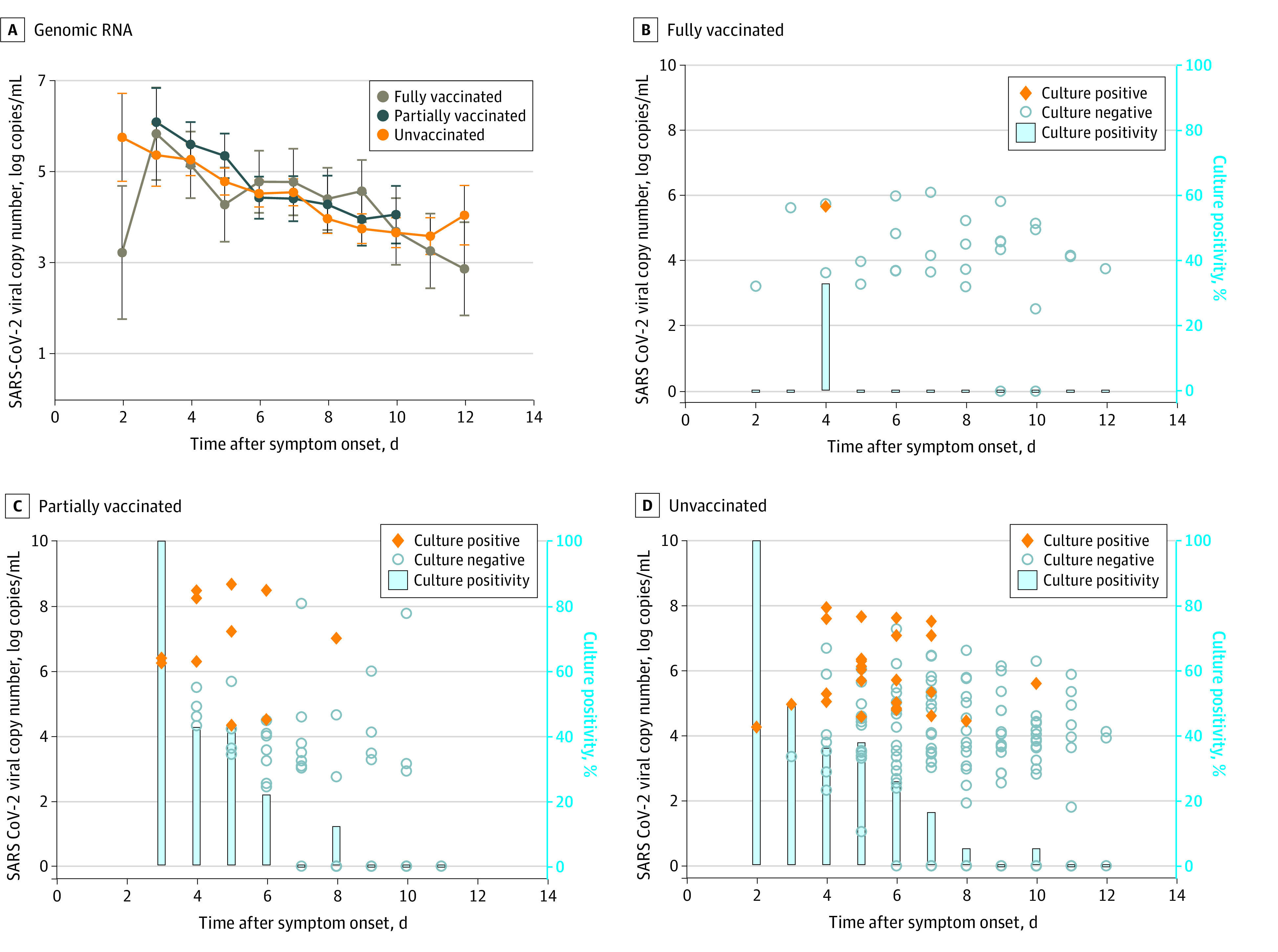
Viral Load Kinetics in Mildly Symptomatic Patients With COVID-19 According to Vaccination Status Kinetics of genomic RNA (mean values with the estimated SEs) in fully vaccinated and partially or nonvaccinated individuals are shown (A). Viral copy number and culture positivity according to the symptom onset date in fully vaccinated individuals (n = 6) (B), partially vaccinated individuals (n = 11) (C), and nonvaccinated individuals (n = 28) (D) are shown.

Of the remaining 45 patients infected with the Delta variant of SARS-CoV-2, 6 (13%) had been fully vaccinated (breakthrough infection), whereas 11 (24%) had been partially vaccinated and 28 (62%) were unvaccinated (nonbreakthrough infection). None had a history of previous infection. Initial genomic viral load was comparable between the fully vaccinated individuals and the partially or unvaccinated individuals (Figure 3A). Genomic viral loads decreased with time in both groups, and the viral loads were similar between the 2 groups at all time points (Figure 3A). However, although fully vaccinated individuals showed viable virus in cell culture until 4 days after symptom onset (Figure 3B), partially vaccinated individuals showed viable virus in cell culture until 8 days after symptom onset and unvaccinated individuals until 10 days after symptom onset (Figure 3C and 3D). The time lag for negative conversion of viral culture was numerically shorter in fully vaccinated individuals (median, 1.75 days; lower limit of the 95% CI, 1 day; upper limit of the 95% CI, not available in our data) than in partially or unvaccinated individuals (median, 4.38 days; 95% CI, 2-7 days; *P* = .42) (eFigure 2 in the Supplement).

## Discussion

In this cohort study, we found that although the initial genomic viral load at diagnosis was similar between fully vaccinated and partially vaccinated or unvaccinated individuals, fully vaccinated individuals had a significantly shorter duration of viable viral shedding and a lower rate of secondary transmission compared with partially vaccinated or unvaccinated individuals. Data regarding transmissibility and viral load kinetics in vaccinated and unvaccinated individuals are scarce. Before the Delta variant became dominant, some studies^5,6^ reported that vaccinated individuals had a lower secondary attack rate than nonvaccinated individuals. However, Singanayagam et al^7^ analyzed the transmission risk for 231 contacts exposed to 162 index cases with the Delta infection in the UK and showed that the secondary attack rate (25%) in household contacts exposed to fully vaccinated index cases was similar to those in unvaccinated index cases (23%).

In contrast to the abovementioned studies, our study showed that in the nosocomial setting, the risk of secondary transmission was significantly lower in the breakthrough infection group than in the nonbreakthrough infection group during the entire study period (3 of 43 [7%] vs 29 of 110 [26%], *P* = .008) and the period after the emergence of the Delta variant (3 of 43 [7%] vs 7 of 28 [25%], *P* = .04), even though the initial Ct value was similar between the 2 groups. Such difference in the secondary attack rate may be partially attributable to differences in the study setting (community [household] vs hospital), but our viral load kinetic results further support the idea that vaccination reduces the transmission risk, because the duration of viable virus shedding was shorter in fully vaccinated individuals than in partially vaccinated or nonvaccinated individuals with Delta variant infection. Similar to our finding, Eyre et al^8^ found that vaccination was associated with reduced onward transmission. Considering the paucity of data on viable viral shedding between vaccinated and unvaccinated individuals infected with the Delta variant, further studies are needed to validate our findings.

No significant association was found between the Ct value at diagnosis and the interval between second vaccination and diagnosis. A previous study^9^ also reported no significant difference in the initial viral load between vaccinated individuals and unvaccinated individuals infected with the Delta variant. Intuitively, a similar level of viral load would confer a comparable potential for viable virus spreading. However, the genomic viral load does not necessarily reflect transmissible viral load. Therefore, we extensively investigated daily saliva viral shedding by cell culture to assess the duration of infectious viral shedding in vaccinated and unvaccinated individuals infected with the Delta variant. Notably, the duration of viable viral shedding after symptom onset was considerably longer in partially or unvaccinated individuals (>1 week) than in fully vaccinated individuals (4 days) (Figure 3). Other studies^10,11^ based on reverse transcriptase PCR result found that individuals with breakthrough infections showed faster clearance than unvaccinated infected individuals. Previous studies^12,13^ consistently reported that although memory B-cell or T-cell responses are maintained more than 6 months after COVID-19 vaccination, the antibody responses against SARS-CoV-2 rapidly wane after COVID-19 vaccination. Because approximately 4 to 5 days are needed for the development of memory B-cell response against SARS-CoV-2, low neutralizing antibody level could ultimately result in a similar early viral load between vaccinated and unvaccinated individuals because of the short latent period of SARS-CoV-2. However, the activating memory B-cell responses in vaccinated individuals might eventually control viable viral shedding more rapidly than that in unvaccinated individuals. In this context, although the number of breakthrough infections was not large enough to detect a statistically significant difference in the viral shedding kinetic study, our findings provide an important implication for public health decision-making on quarantine or isolation policies and epidemiologic investigation of breakthrough infections. Further discussion is available in the eDiscussion in the Supplement.

### Limitations

This study has several limitations. First, the cohort 1 study spanned the pre-Delta and post-Delta eras, with most of the nonbreakthrough infection cases occurring in the pre-Delta period. Therefore, some may argue that this full epidemic period with different circulating variants might introduce significant bias. However, because the Delta variant has higher transmission potential than the original strain,^14^ our study setting could have resulted in a bias toward the null. Furthermore, the subgroup analysis on patients recruited after the emergence of the Delta variant in South Korea revealed similar findings.

Second, considering that breakthrough infection was more common in HCWs than in non-HCWs, HCWs might have been diagnosed with COVID-19 earlier and had less chance to spread the virus. In addition, we analyzed only nosocomial transmission; we did not analyze secondary transmission in the households of HCWs (mostly vaccinated people). We evaluated all in-hospital contacts for those inpatients and caregivers who were mostly unvaccinated. Therefore, this approach could bias the differences in secondary attack rates. However, the stratified analysis between HCWs and non-HCWs showed a similar trend toward higher secondary attack rates in unvaccinated individuals than in fully vaccinated individuals among HCWs and non-HCWs, albeit without statistical significance because of the small sample size (eTable 3 in the Supplement).

Third, we used daily saliva specimens instead of nasopharyngeal (NP) swab samples for the viral kinetics study to facilitate dense sampling. Variability in Ct values, even with the same day, was shown with saliva specimens,^15^ and the debate about viral shedding at different sites continues with Omicron variant surges. In addition, the SARS-CoV-2 viral load was lower in saliva samples than in NP swab samples,^16^ but a meta-analysis^17^ revealed that the sensitivity of the 2 types of samples was comparable. Although NP swab samples may have been useful in studying the viral shedding kinetics in further detail, obtaining daily NP swab samples was logistically challenging; therefore, our approach using daily saliva samples might be the best alternative for understanding the viral shedding kinetics of SARS-CoV-2.

Fourth, 10% of vaccinated individuals and 20% of unvaccinated individuals did not have Ct value results, which may be a selection bias. However, the viral kinetic study in cohort 2 revealed the same findings that initial viral load was similar between breakthrough and nonbreakthrough infection (Figure 3A).

Fifth, we did not analyze the waning immunity of vaccine effectiveness in this study. The median time from second vaccination to diagnosis of breakthrough infection was 98 days, and the follow-up period was short for observing whether vaccine effectiveness against transmission waned. Recently, Eyre et al^8^ found that protection against onward transmission waned during the 3-month period after the second vaccination. Further studies are needed to determine whether vaccine effectiveness against transmission waned.

Sixth, given the low number of vaccinated individuals (n = 6) in the viral kinetic study, those findings may need more validation. The sensitivity analysis of leave-one-out cross-validation revealed that there were not substantial potential outliers. A further large study to validate our findings is needed. Despite this limitation, our data provide valuable insights into the transmissibility among vaccinated individuals compared with unvaccinated individuals given that few data are available on the effect of vaccination on facilitating viable viral clearance.

## Conclusions

In this cohort study of HCWs, inpatients, guardians, and participants in a community facility, although fully vaccinated individuals had similar levels of genomic viral load at diagnosis as unvaccinated individuals, fully vaccinated individuals had a notably shorter duration of viable viral shedding and a significantly lower secondary attack rate. Data from this study provide important evidence that despite the possibility of breakthrough infections, COVID-19 vaccinations remain critically useful for controlling the spread of SARS-CoV-2.

## Notes

Supplement. **eTable 1.** Primers and Probes Used for Real-time RT-PCR Assay for the Detection of N Gene and S Gene of SARS-CoV-2 **eTable 2.** Characteristics of Individuals With COVID-19 Infection in Fully Vaccinated, Partially Vaccinated, and Non-vaccinated Individuals in Cohort 1 **eTable 3.** Subgroup Analysis for Secondary Transmission Rate in Healthcare Workers (HCWs) and Nonhealthcare Workers (Non-HCWs) **eMethods.** Supplementary Methods **eFigure 1.** Flowchart of Patients in Cohort 2 **eFigure 2.** Estimation of the Proportion of the Viral Shedding in Terms of Viral Culture **eDiscussion.** Supplementary Discussion
eReferences
