# Supplementary material for: Transmission and Infectious SARS-CoV-2 Shedding Kinetics in Vaccinated and Unvaccinated Individuals
Source: JAMA Netw Open. 2022 May 24;5(5):e2213606. doi: 10.1001/jamanetworkopen.2022.13606 (PMC9131744; doi:10.1001/jamanetworkopen.2022.13606)
Supplement: Supplement. — eTable 1. Primers and Probes Used for Real-time RT-PCR Assay for the Detection of N Gene and S Gene of SARS-CoV-2 eTable 2. Characteristics of Individuals With COVID-19 Infection in Fully Vaccinated, Partially Vaccinated, and Non-vaccinated Individuals in Cohort 1 eTable 3. Subgroup Analysis for Secondary Transmission Rate in Healthcare Workers (HCWs) and Nonhealthcare Workers (Non-HCWs) eMethods. Supplementary Methods eFigure 1. Flowchart of Patients in Cohort 2 eFigure 2. Estimation of the Proportion of the Viral Shedding in Terms of Viral Culture eDiscussion. Supplementary Discussion eReferences [file jamanetwopen-e2213606-s001.pdf]

Supplementary Online Content

Jung J, Kim JY, Park H, et al. Transmission and infectious SARS-CoV-2 shedding kinetics in vaccinated and unvaccinated individuals. *JAMA Netw Open*. 2022;5(5):e2213606. doi:10.1001/jamanetworkopen.2022.13606

eTable 1. Primers and Probes Used for Real-time RT-PCR Assay for the Detection of N Gene and S Gene of SARS-CoV-2

eTable 2. Characteristics of Individuals With COVID-19 Infection in Fully Vaccinated, Partially Vaccinated, and Non-vaccinated Individuals in Cohort 1

eTable 3. Subgroup Analysis for Secondary Transmission Rate in Healthcare Workers (HCWs) and Nonhealthcare Workers (Non-HCWs)

eMethods. Supplementary Methods

eFigure 1. Flowchart of Patients in Cohort 2

eFigure 2. Estimation of the Proportion of the Viral Shedding in Terms of Viral Culture

eDiscussion. Supplementary Discussion

eReferences

This supplementary material has been provided by the authors to give readers additional information about their work.

© 2022 Jung J et al. *JAMA Network Open.*

eTable 1. Primers and probes used for real-time RT-PCR assay for the detection of N gene and S gene of SARS-CoV-2

Target (Accession #)	Name	Location	Sequence	Modification
N gene (NC_045512)	NF	29356	AACATTCCCACCAACAGAGC	
	NR	29529	GCCTGAGTTGAGTCAGCACT	
	NP	29462	GCTGATGAAACTCAAGCCTTACCGCA	5’Cy5, 3’BHQ2
S gene (NC_045512)	SF	21624	GAACTCAATTACCCCCTGCAT	
	SR	21787	ACCATTGGTCCCAGAGACAT	
	SP	21657	TCACACGTGGTGTTTATTACCCTGACA	5’FAM, 3’BHQ1
Internal control (NC_000007.14)	BAF	1670	ACTAACACTGGCTCGTGTGA	
	BAR	1774	CTTGGGATGGGGAGTCTGTT	
	BAP	1700	AGGCTGGTGTAAAGCGGCCTTGG	5’HEX, 3’BHQ1

Footnote. The samples were considered positive for SARS-CoV-2 genomic RNA detection if both N and S genes were positive as well as the positive internal control.

© 2022 Jung J et al. *JAMA Network Open.*

eTable 2. Characteristics of individuals with COVID-19 infection in fully vaccinated, partially vaccinated, and non-vaccinated individuals in cohort 1.

	Fully vaccinated individuals (n=50)	Partially vaccinated individuals (n=14)[a]	Non-vaccinated individuals (n=109)	P value
**Age, years**	46 (31–59)	57 (32-63)	46 (44-58)	0.46
**Female**	27 (54)	10 (71)	63 (58)	0.51
**Healthcare worker**	42 (84)	7 (50)	57 (52)	<0.001
**Inpatient**	7 (14)	5 (36)	28 (26)	0.14
**Guardian or caregiver**	1 (2)	2 (14)	24 (22)	0.005
**Days from second vaccination to diagnosis**	98 (57–143)	N/A	N/A	N/A
**Type of COVID-19 vaccine**				
ChAdOx-nCoV-1	40 (80)	9 (64)	-	0.29
BNT162b2	5 (10)	2 (14)	-	0.64
mRNA-1273	2 (4)	3 (21)	-	0.09
Heterologous[*]	2 (4)	0 (0)	-	>0.99
Other[#]	1 (2)	0 (0)	-	>0.99
**Symptomatic at diagnosis**	38 (76)	7 (50)	75 (69)	0.17
**Days from symptom onset to diagnosis**	1 (0–1)	1 (1-2)	1 (0-3)	0.13
**Ct value at diagnosis**	19 (16–24)[b]	25 (18-32)[c]	19 (15-28)[d]	0.16
**Nosocomial secondary transmission[d], % (n/N)**	7% (3/43)	20% (2/10)	27% (27/100)	0.03

Values are median (interquartile range) or n (%) unless indicated otherwise. Categorical variables were analyzed using the chi-squared test, and continous variables were analyzed using Kruskal-Wallis test.

[a]Partially vaccination was defined as from 2 weeks after the first vaccination to within 2 weeks after the second vaccination.

[b]including 45 individuals who underwent SARS-CoV-2 testing at our hospital; Ct values were unavailable in the remaining 5 individuals

[c]including 12 individuals who underwent SARS-CoV-2 testing at our hospital; Ct values were unavailable in the remaining 2 individuals

[d]including 91 individuals who underwent SARS-CoV-2 testing at our hospital; Ct values were unavailable in the remaining 18 individuals

[*]ChAdOx1 nCoV-19 followed by BNT162b2

[#]rAD26 and rAd5 vector-based vaccine

© 2022 Jung J et al. *JAMA Network Open.*

eTable 3. Subgroup analysis for secondary attack rate in healthcare workers (HCWs) and non-healthcare workers (Non-HCWs)

	**Breakthrough infection**	**Non-breakthrough infection**	***P* value**
**Nosocomial secondary transmission during the total study period (%)**			
HCWs	5% (2/37)	16% (9/58)	.19
Non-HCWs	17% (1/6)	38% (20/52)	.40
**Nosocomial secondary transmission after the emergence of the delta variant (%)**			
HCWs	5% (2/37)	19% (3/16)	.15
Non-HCWs	17% (1/6)	33% (4/12)	.13

© 2022 Jung J et al. *JAMA Network Open.*

eMethods. Supplementary Methods

Cohort 1: Epidemiologic investigation, hospital policy for screening for COVID-19 cases, and vaccination program for healthcare workers at the hospital

Before the emergence of the omicron wave in South Korea, the Korean government extensively traced close contacts with confirmed COVID-19 patients by mandatory QR code check-in system, and sent the text messages to close contacts to encourage COVID-19 testing. In addition, the infection control team in our hospital meticulously traced close contacts of all HCWs, inpatients, and caregivers with confirmed COVID-19 patients. So, during the study period, we included all HCWs who were diagnosed with SARS-CoV-2 infection as well as all inpatients and caregivers who developed COVID-19 during the hospital stay or diagnosed COVID-19 outside soon after discharge; the hospitalization period was included in their infectious period (≤2 days before the symptom onset day in symptomatic cases or ≤2 days before the diagnosis day in cases of asymptomatic cases). Among these cases, secondary transmitted cases were identified by the epidemiologic investigations.

All inpatients and their caregivers were subjected to daily screening with temperature monitoring for symptoms and epidemiologic links; if they showed any symptoms or epidemiologic links, they underwent SARS-CoV-2 PCR testing. Since April 2020, we have implemented a universal pre-admission screening policy for SARS-CoV-2 using RT-PCR in nasopharyngeal (NP) swab specimens. Then, we expanded the universal screening to hospital day 4 for all inpatients in December 2020 in response to the 3rd wave in South Korea, and again expanded the screening to hospital day 8 in August 2021 and hospital day 14 in October 2021 in response to the 4th wave. Guardians or caregivers of the inpatients also need to undergo universal SARS-CoV-2 testing before residence in the ward and residence days 4, 8, and 14. As for the HCWs at Asan Medical Center, all HCWs undergo mandatory daily

© 2022 Jung J et al. *JAMA Network Open.*

monitoring of symptoms and undergo PCR testing in cases of symptoms or epidemiologic links.

Nosocomial secondary infection was defined as cases of SARS-CoV-2 acquisition in hospital with epidemiologic links inside of the hospital and without definite epidemiologic links outside the hospital regardless of hospital stay. Individuals who had negative SARS-CoV-2 PCR results at the time of the start of quarantine did not have any chance for further transmission. So, individuals who had negative SARS-CoV-2 PCR results at the time of the start of quarantine, or were found to have been exposed to SARS-CoV-2 and quarantined in a single room (for inpatient and caregiver) or at home (for HCWs) were excluded from the denominator in the transmission analysis as they could not transmit the virus in the hospital.

From March 5, 2021, HCWs began receiving vaccinations at the COVID-19 vaccine center in our hospital. Four COVID-19 vaccine regimens have been used in our hospital during the study period: ChAdOx1 nCoV-19, BNT162b2, mRNA-1273, and heterologous vaccination with ChAdOx1 nCoV-19 followed by BNT162b2. The intervals between the two doses of ChAdOx1 nCoV-19, BNT162b2, and mRNA-1273 vaccines were 12 weeks, 3 weeks, and 4 weeks, respectively.

Identification of the delta variant

The presence of the delta variant in saliva samples collected from cohort 2 was assessed as follows. The collected saliva samples were inactivated at 65℃ for 30 min in a special negative pressure laboratory. Genomic viral RNA was extracted from the specimens using a QIAamp viral RNA Mini kit (Qiagen Inc., Hilden, Germany). The variants were classified by double-multiplex real-time RT-PCR assay using a PowerChek SARS-CoV-2 S-gene Mutation detection kit (Kogene biotech co. Ltd, Seoul, South Korea) according to the manufacturer’s instructions. PCR amplifications were performed using AB 7500 Fast Real-

© 2022 Jung J et al. *JAMA Network Open.*

Time PCR System (Applied Biosystems, Foster City, CA, USA) and the delta variant was identified as positive results of both P681R and L452R mutations.

Measurement of genomic viral load by real-time RT-PCR assay

To determine the SARS-CoV-2 genomic viral RNA copy number, multiplex real-time RT-PCR assays targeting the S and N genes were developed (eTable 1). Multiplex RT-PCR assay mix (20 μL) contained 4 μL of 5X master mix (LightCycler Multiplex RNA Virus Master, Roche, Basel, Switzerland), 0.1 μL of 200X enzyme mix, 500 nM of each S and N gene primer, 200 nM of each S and N gene probe, 250 nM of internal control primers, 100 nM of internal control probes, and 5 μL of extracted RNA or in vitro-synthesized control RNA. PCR amplification was performed with a LightCycler 96 system (Roche) in the following conditions: reverse transcription at 50℃ for 10 min, initial denaturation at 95℃ for 5 min, 45 cycles of 2-step amplification, denaturation at 95℃ for 10 s, and final extension at 60℃ for 30 s. To generate calibration curves, serial dilutions from 10^7 to 5 copies/μL of synthetic control RNA were assayed in six independent sets of reactions (Supplementary Figure 4). The detection limit of this assay was 5 copies/reaction (2.6 log copies/ml of specimen) and viral copy numbers were determined by plotting CT values against log copies/reaction.

© 2022 Jung J et al. *JAMA Network Open.*

Cell culture

Culture-based isolation of SARS-CoV-2 from saliva specimens that revealed positive genomic RNA results was performed by a plaque assay in a Biosafety Level 3 laboratory at Korea University College of Medicine (Seoul, South Korea). Briefly, Vero cells were cultured at 9×10^5 cells/well in 6-well plates for 24 h, and specimens were serially diluted 10-fold using PBS; then, 200 µl of each diluted sample were inoculated into cells and incubated for 1 h (37°C, 5% CO_2) with rocking every 15 min, and overlaid with 2 mL of Dulbecco's Modified Eagle Medium/Nutrient Mixture F12 (DMEM/F-12) medium containing 0.6% oxoid agar. Viral plaque formation was visualized by crystal violet staining after 72 h of incubation in a 5% CO_2 incubator at 37°C.

© 2022 Jung J et al. *JAMA Network Open.*

eFigure 1. Flowchart of patients in cohort 2.

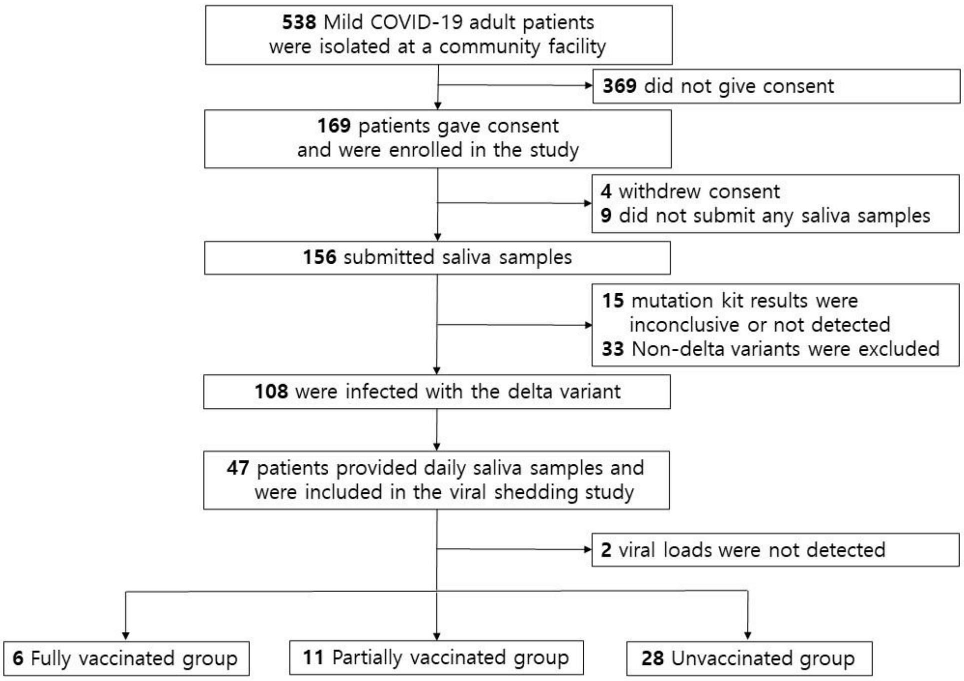

© 2022 Jung J et al. *JAMA Network Open.*

eFigure 2. Estimation of the proportion of the viral shedding in terms of viral culture. Orange rectangles indicate the proportions of censored data, and gray lines represent the 95% confidential interval of the fitted distributions. Red horizontal lines indicate the 95% CI of the time to negative viral culture or subgenomic RNA for the median number of the patients.

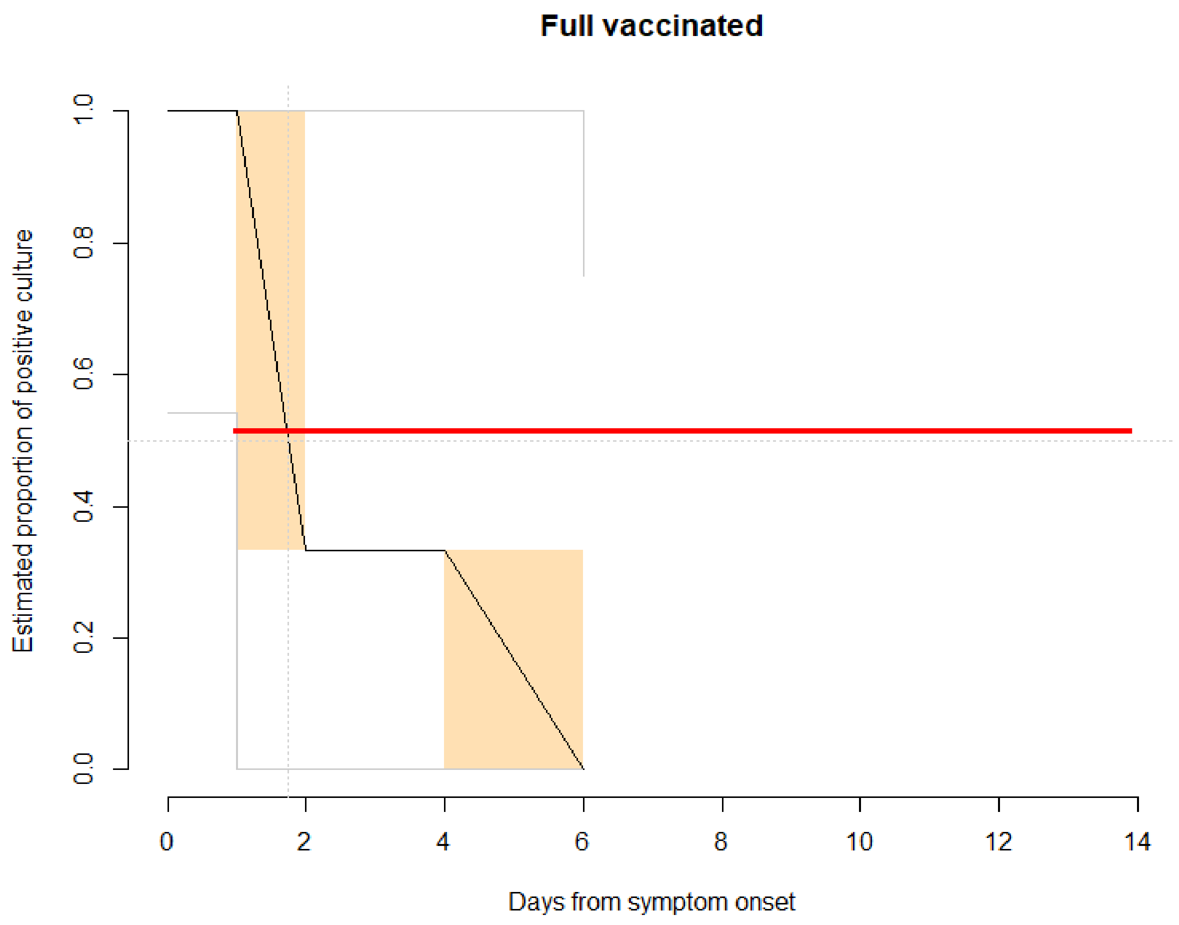

© 2022 Jung J et al. *JAMA Network Open.*

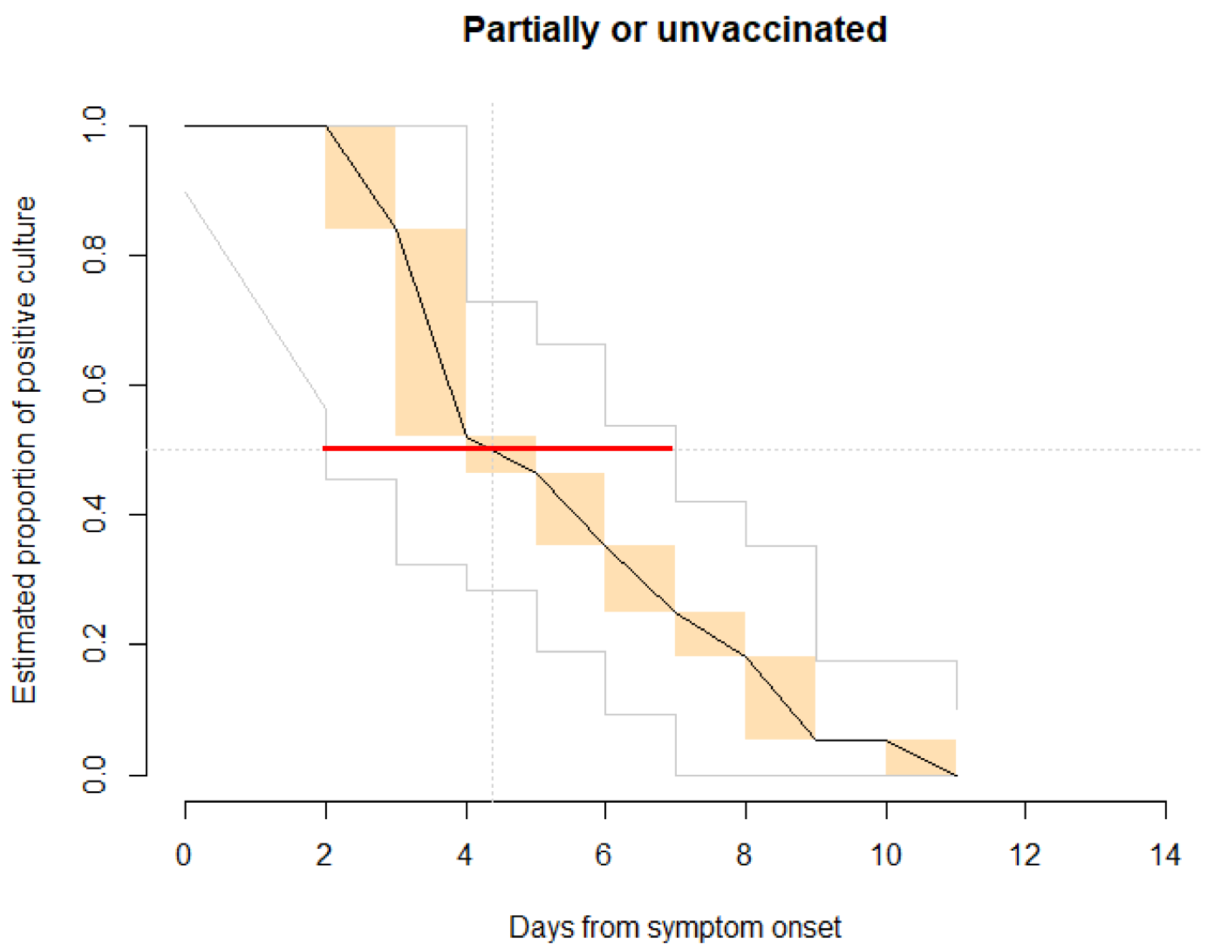

© 2022 Jung J et al. *JAMA Network Open.*

eDiscussion. Supplementary Discussion

Our study was performed before the Omicron variant emerged, and we only analyzed the individuals who were fully vaccinated which was defined as receipt of two dose vaccination and non-fully vaccinated. Vaccine effectiveness of 2 doses of mRNA vaccine against Omicron variant is less than 50%,[1,2] and fully vaccination or up-to-date vaccination may be considered as 3 doses of vaccine in the Omicron era. Thus, further study is needed regarding the differences in transmission and viral load kinetics in fully vaccinated (completion of 3 doses) and non-fully vaccinated individuals in Omicron predominant period.

Previous SARS-CoV-2 infection may protect against re-infection, but we did not perform serologic test before the breakthrough infection. However, we interviewed all COVID-19 patients about the history of past infection, and no had history of past infection. From 2020 to 2021, the incidence of SARS-CoV-2 infection was low in South Korea, and as of December 31, 2021, the number of cumulative cases was 630,838 (1,219 per 100,000 persons) in South Korea. In addition, national seroprevalence (seropositivity for N protein) by natural infection which was investigated by Korea Diseases Control and Prevention Agency from July 15 to October 22, 2021 was 0.54% (95% CI, 0.2-0.9%) (personal communication from Dr. H-C Jang). So, this bias may not substantially affect our main findings.

© 2022 Jung J et al. *JAMA Network Open.*

eReferences

1. Tseng HF, Ackerson BK, Luo Y, et al. Effectiveness of mRNA-1273 against SARS-CoV-2 Omicron and Delta variants. Nat Med. 2022. E-pub ahead of print.

2. Accorsi EK, Britton A, Fleming-Dutra KE, et al. Association Between 3 Doses of mRNA COVID-19 Vaccine and Symptomatic Infection Caused by the SARS-CoV-2 Omicron and Delta Variants. JAMA. 2022;327(7):639-51.

© 2022 Jung J et al. *JAMA Network Open.*
